# Patient and aneurysm characteristics in familial intracranial aneurysms. A systematic review and meta-analysis

**DOI:** 10.1371/journal.pone.0213372

**Published:** 2019-04-08

**Authors:** Emma M. H. Slot, Gabriel J. E. Rinkel, Ale Algra, Ynte M. Ruigrok

**Affiliations:** 1 Brain Center Rudolf Magnus, Department of Neurology and Neurosurgery, University Medical Center, Utrecht, the Netherlands; 2 Julius Center for Health Sciences and Primary Care, University Medical Center, Utrecht, the Netherlands; Universitatsklinikum Freiburg, GERMANY

## Abstract

**Background and purpose:**

Patient and aneurysm characteristics have been reported to differ between patients with familial and non-familial intracranial aneurysms (IAs), although results are inconsistent. We systematically reviewed and meta-analyzed the literature to identify and quantify patient- and aneurysm characteristics associated with familial IAs.

**Methods:**

We searched PubMed and EMBASE for case-control and cohort studies comparing patient- and aneurysm characteristics between familial and non-familial IAs. Two observers independently assessed study eligibility and appraised quality with the Newcastle Ottawa Scale. With univariable weighted linear regression analysis we calculated β-coefficients with corresponding 95% confidence intervals (CIs) for ruptured and unruptured IAs combined and for ruptured IAs only. Heterogeneity was assessed with Higgins I^2^.

**Results:**

A total of 15 articles were included in the meta-analysis in which 16,346 patients were analyzed with a total of 14,225 IAs. For ruptured and unruptured IAs combined, multiple IAs were more prevalent in familial (28.5%) than in non-familial IAs (20.4%; β = 0.10, 95% CI, 0.04 to 0.16; I^2^ 0%). For ruptured IAs only, in familial patients IAs were more prevalent on the middle cerebral artery (41.1% versus 29.5%; β = 0.12, 95% CI, 0.01 to 0.24; I^2^ 12%) and ruptured at a younger age (46.5 years versus 50.8 years; β = -5.00, 95% CI, -9.31 to -0.69; I^2^ 98%) than in non-familial patients. No significant differences were found for the proportion of women, size of the aneurysm at time of rupture, smoking or hypertension.

**Conclusion:**

These results suggest that characteristics of familial and non-familial IAs show considerable overlap, yet differ on specific aspects. However, results for age at rupture showed considerable heterogeneity. These findings should be taken into consideration for future etiological research into IAs.

## Introduction

Family history is the strongest risk factor for aneurysmal subarachnoid hemorrhage (aSAH) caused by a ruptured intracranial aneurysm (IA) and 10% of aSAH patients have a positive family history for aSAH [1,2]. Differences in clinical characteristics have been described for patients with familial IAs compared to those with non-familial IAs, although the reported results are inconsistent, for example for proportion of women [3–10], age at SAH onset [3,4, 9, 11–13], site [4–7, 9–12], size [11, 13, 14] and multiplicity of aneurysms [3, 5, 7, 9–14] and outcome after SAH [15–17]. A clear understanding of etiological differences between familial and non-familial IA is essential for clinical practice of IA patients and in future etiologic research on IA.

This systematic review and meta-analysis aims to identify and quantify patient- and aneurysm-specific characteristics associated with familial IAs as compared to non-familial IAs.

## Methods

### Study design

The authors followed the PRISMA guidelines for this systematic review and meta-analysis. This study is not subject to review by the UMC Utrecht’s Ethics Committee as it does not involve acquisition of new individual patient data.

### Search strategy and selection criteria

We searched PubMed and EMBASE until December 2018 for case-control and cohort studies comparing characteristics of familial and non-familial IA with the search terms “Familial” OR “Family” OR “Families” OR “Heritable” OR “Heredity” OR “First-degree Relatives” OR “Genetic” AND “Intracranial Aneurysm” OR “Cerebral Aneurysm” OR “Subarachnoid Hemorrhage” OR “Subarachnoid Haemorrhage” and relevant MeSH terms. One author (E.M.H.S.) identified relevant articles from the database search based on title and abstract. We included studies on familial IAs using a broad definition of at least two relatives with ruptured or unruptured IAs. Studies were excluded if they were 1. case reports; 2. written in other languages than English, Dutch, Spanish, French and Italian; 3. published before 1966. Patients with IA and Autosomal Polycystic Kidney Disease or connective tissue disorders, such as Ehlers-Danlos disease were excluded. Two authors (E.M.H.S. and Y.M.R.) independently screened all remaining abstracts and full-text articles on their eligibility for inclusion. The reference lists of relevant articles were crosschecked for further relevant studies until no further publications were found.

### Data-extraction

Two authors (E.M.H.S. and Y.M.R.) independently extracted crude data and effect estimates if available for patient- and aneurysm-specific characteristics associated with familial versus non-familial IAs on a standardized data-extraction form. The following patient-specific characteristics were collected: age at time of aneurysm rupture, sex, smoking, hypertension, clinical condition on admission and outcome at or after discharge. We extracted data for the following aneurysm-specific characteristics: multiplicity of IAs, size of the aneurysm at time of rupture, risk of rupture and aneurysm location. Multiplicity was extracted as a dichotomous variable. Aneurysm locations were divided into four categories: anterior cerebral artery, including anterior communicating artery and pericallosal artery (ACA), middle cerebral artery (MCA), internal carotid artery, including posterior communicating artery (ICA) and vertebrobasilar artery (VBA). In addition, we collected per study data on 1. study design; 2.definition of familial IAs used; 3. total number of patients, including total number familial IA patients; 4. total number of IAs, including total number of familial IAs; 5. type of IA studied, i.e. ruptured, unruptured or both; 6. nationality of the patient population; 7. method used for diagnosing familial IAs (using interview, medical records or screening with imaging or a combination). The quality of the included articles was individually assessed by each of the two reviewers according to the Newcastle Ottawa Scale for case-control studies [18]. Studies with a Newcastle Ottawa Scale of more than five out of nine points were considered high quality.

### Statistical analysis

All patient- and aneurysm-specific characteristics were analyzed by performing a univariable weighted linear regression analysis to calculate the β-coefficients (B) with 95% confidence intervals (CIs). The regression was weighted based on the number of IAs in case of aneurysm-specific characteristics and on the number of patients in case of patient-specific characteristics. For dichotomous variables proportions were calculated based on the number of patient or aneurysm measurements for the variable. We intended to perform a separate analysis for ruptured IAs and one for unruptured IAs. Since many studies included in our analysis did not make a clear distinction between these two groups of IAs we performed the following two analyses instead: 1. including both ruptured and unruptured IAs and 2. including ruptured IAs only. Additionally, we performed the following sensitivity analyses: first, we only included studies defining familial IAs as at least two first-degree relatives with IAs, as this is the strictest definition for familial IAs used in the literature; second, we only included studies of high quality (>5 points on the Newcastle Ottawa Scale); third we excluded Finnish and Inuit populations, as these may represent different clinical subtypes. Heterogeneity of the data across studies was assessed through Higgins I^2^ using the most adjusted effect estimates reported in the original publications [19]. If no effect estimates were available we used crude data to calculate the odds ratios (ORs) and 95% CIs for dichotomous variables. The mean difference and standard deviations were retrieved if available to calculate I^2^ for continuous variables. For those studies that did not report the standard deviations we used the mean standard deviation calculated from the studies that did report the standard deviation for that specific variable. We defined I^2^ ≤ 60% as little to moderate heterogeneity and I^2^>60% as substantial to considerable heterogeneity.

## Results

**Fig 1:**
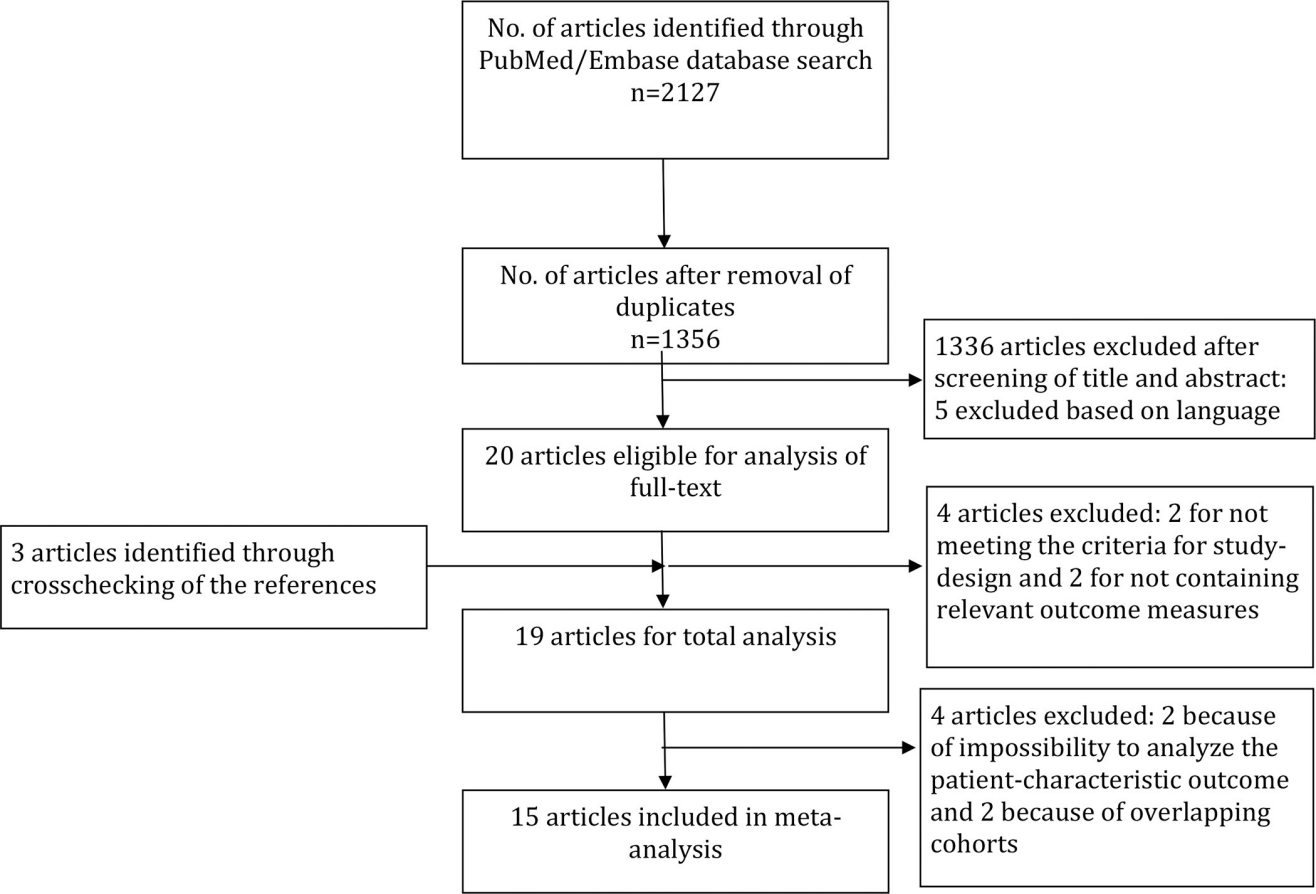
Flowchart of the study selection process.

The database search yielded 2,092 articles of which 15 articles were eligible for our meta-analysis (Fig 1). The vast majority of manuscripts was excluded based on screening of title or abstract because they did not compare patient or aneurysm characteristics between familial and non-familial patients. Five articles were excluded because they were written in languages than those defined in our inclusion criteria. Two of these could be excluded based on their English written abstract. We intended to analyze the characteristics condition on admission and outcome at or after discharge in our meta-analysis. However, since the studies reporting on these characteristics used different outcome measures, which were also measured at different time points [15–17] we had to exclude these characteristics from further analysis (Fig 1). We identified a single study showing a higher risk of rupture of familial IAs compared to non-familial IAs [20]. Therefore, we were not able to include the characteristic risk of rupture in the meta-analysis either. We included 15 articles in the meta-analysis. For patient characteristics 16,346 patients were analyzed of whom 2,359 were familial IA patients. For aneurysm characteristics 14,225 IAs of which 2,275 were familial ones. Table 1 provides an overview of the study characteristics and included patient-populations. The different definitions of familial IAs used and the different ways of how the familial IAs were diagnosed in the included studies are reported in S1 Table. Eight out of the 15 included studies were considered as high quality studies (S2 Table) [2,3, 7, 9, 10, 13, 14, 20].

**Table 1:** Overview of study characteristics.

First author	Nationality	Study design	No. of all patients	No. of familial IA patients only	No. of all IAs	No. of familial IA only	Rupture status
**Lozano** [11]	Canadian	NCCC with controls from different studies	3039^a^	177	2928^b^	243	Both
**Norrgard** [10]	Swedish	RCC	579	46	335	59	Ruptured
**Ronkainen** [3]	Finnish	RCC	1150	167	1511	215	Ruptured
**Bromberg** [9]	Dutch	PCC	144	19	136^c^	14^c^	Ruptured
**Schievink** [6]	North-American	RCC	76	15	76^c^	15^c^	Ruptured
**LeBlanc** [4]	Canadian	PCC with controls from a different study	2657^d^	30	2379^c^	30^c^	Ruptured
**Mathieu** [7]	Canadian	RCC	502	144	502^c^	144^c^	Ruptured
**Ronkainen** [21]^e^	Finnish	RCC	1357	120	1853	120	Ruptured
**Connolly** [8]	Canadian	RCC	785	54	-	-	Both
**Lindgaard** [12]	Danish and Inuit (separate)	RCC	1157	79	1089^c^	57^c^	Ruptured
**Ruigrok** [14]	Dutch	RCC	146	58	185	86	Ruptured
**Lee** [22]	Korean	RCC	1128	12	1128^c^	12^c^	Ruptured
**Broderick** [20]	American, New-Zealand, Australian	Retrospective cohort and prospective cohort with separate CC	2874	611	-	-	Both
**Huttunen** [13]	Finnish	RCC from prospective data-base	1770	316	2066^c^	489^c^	Both (separate)
**Mackey** [5]	American, Canadian, Australian and New Zealand, European	RCC	2930	511	4175	791	Unruptured

IA = intracranial aneurysm,NCCC = non-consecutive case-control, RCC = retrospective case-control, PCC = prospective case-control, CC = case-control

^a^Including 2 cohorts of non-familial IA patients from different studies: 2627 patients [23] and 235 patients [24]

^b^Including 2 cohorts of non-familial IAs from different studies: 2627 patients [23] and 58 patients [25]

^c^Total number of IAs not reported, number represents the number of IAs available for analysis

^d^Including non-familial IA patients from a different study: 2627 patients [23]

^e^This cohort is largely overlapping with Ronkainen et al. 1995 [3]

### Ruptured and unruptured aneurysms

Table 2 provides an overview of the different patient- and aneurysm-specific characteristics assessed for the joint analysis of ruptured and unruptured IAs.

**Table 2:** Patient and aneurysm-specific characteristics of familial intracranial aneurysms as compared to non-familial intracranial aneurysms for analysis of ruptured and unruptured aneurysms together.

Characteristic	Familial IAs	Non-familial IAs	Β^a^	95% CI	Heterogeneity I^2^ (%)
**Women (%)**	62.9	60.6	0.03	-0.07 to 0.14	70
**Smoking (%)**	55.7	52.6	-0.06	-0.89 to 0.78	72
**Hypertension (%)**	47.5	54.9	0.01	-0.46 to 0.48	95
**Multiplicity (%)**	28.5	20.4	0.10	0.04 to 0.16	0
**ACA (%)**	24.3	33.7	-0.06	-0.22 to 0.10	60
**ICA (%)**	26.1	24.5	-0.031	-0.14 to 0.08	11
**MCA (%)**	38.1	27.9	0.047	-0.05 to 0.15	35
**VBA (%)**	5.5	7.2	-0.0.35	-0.04 to 0.04	0

IA = intracranial aneurysm, 95% CI = 95% confidence interval, ACA = anterior cerebral artery, including the anterior communicating artery and pericallosal artery, MCA = medial cerebral artery, ICA = internal carotid artery, VBA = vertebrobasilar artery

^a^beta calculated with weighted linear regression

### Patient-specific characteristics

The mean proportion of women in the familial IA group was 62.9%, which is comparable to the percentage of women of 60.6% in the non-familial IA group (β = 0.03, 95% CI -0.07 to 0.14). Between familial IA and non-familial IA patients no differences were observed in the prevalence of the risk factors smoking (55.7% as compared to 52.6%; β = -0.06, 95% CI -0.89 to 0.76, p = 0.80) and hypertension (47.5% as compared to 54.9%; β = 0.01, 95% CI -0.46 to 0.48, p = 0.96). Substantial to considerable heterogeneity was found for percentage of women, smoking and hypertension (Table 2).

### Aneurysm-specific characteristics

Multiple IAs were found more often in familial than non-familial IA patients (28.5% compared to 20.4%; β = 0.10; 95% CI, 0.04 to 0.16) (S3 File). The proportion of IAs located at the MCA tended to be higher in familial IAs (38.1%) compared with non-familial IAs (27.9%), but this difference did not yield statistical significance (β = 0.05, 95% CI, -0.05 to 0.15). For the ACA location proportions were 24.3% for familial and 33.7% for non-familial IA. This difference was not statistically significant (β = -0.06, 95% CI -0.22 to 0.98). For the other locations proportions were similar for familial and non-familial IA (Table 2). We found little to moderate heterogeneity between effect estimates for the aneurysm-specific characteristics multiplicity of IAs and the aneurysm locations ICA, MCA and VBA (Table 2). Substantial to considerable heterogeneity was found for aneurysm location ACA (Table 2).

### Ruptured aneurysms only

The different patient- and aneurysm-specific characteristics assessed for the analysis on ruptured IAs only are shown in Table 3.

**Table 3:** Patient and aneurysm-specific characteristics of familial intracranial aneurysms as compared to non-familial intracranial aneurysms for analysis of ruptured aneurysms only.

Characteristic	Familial IAs	Non-familial IAs	Β^a^	95% CI	Heterogeneity I^2^ (%)
**Women (%)**	62.3	59.0	0.00	-0.12 to 0.11	66
**Mean age at rupture (yrs.)**	46.5	50.8	-5.00	-9.31 to -0.69	98
**Mean size at rupture (mm)**	12.9	14.7	-5.52	-19.17 to 8.13	99
**ACA (%)**	27.7	35.9	-0.01	-0.18 to 0.16	58
**ICA (%)**	24.3	22.8	-0.05	-0.18 to 0.08	0
**MCA (%)**	41.1	29.5	0.12	0.01 to 0.24	12
**VBA (%)**	5.3	7.5	-0.01	-0.06 to 0.04	0

IA = intracranial aneurysm, 95% CI = 95% confidence interval, ACA = anterior cerebral artery, including the anterior communicating artery and pericallosal artery, MCA = medial cerebral artery, ICA = internal carotid artery, VBA = vertebrobasilar artery

^a^beta calculated with weighted linear regression

### Patient-specific characteristics

The proportion of women was 62.3% for familial and 59.0% for non-familial IA (β = -0.003, 95% CI -0.12 to 0.11). Age at time of rupture of the IA was lower in familial IA than in non-familial IA patients (46.5 years versus 50.8 years; β = -5.00, 95% CI -9.31 to -0.69) (S3 File). Substantial heterogeneity was found for both the proportion of women and the age at time of rupture (Table 3).

### Aneurysm-specific characteristics

The mean size of the IA at time of rupture tended to be lower in the familial IA group (12.9 mm) compared with that in the non-familial IA group (14.7 mm), however this difference was not statistically significant (β = -5.52, 95% CI -19.17 to 8.13). On comparing aneurysm location between familial IA and non-familial IA patients we found a higher prevalence of IAs originating from the MCA in the familial IA group (41.1% versus 29.5%; β = 0.12, 95% CI 0.01 to 0.24) (S3 File). A lower prevalence of ACA IAs was found among familial IA patients (27.7%) as compared to non-familial IA patients (35.9%). Yet, this difference was not statistically significant (β = -0.007, 95% CI -0.18 to 0.16). For the remaining aneurysm locations comparable prevalence rates between familial IA and non-familial IA patients were found (Table 3). The aneurysm locations ACA, ICA, MCA and VBA showed little to moderate heterogeneity (Table 3). Substantial heterogeneity was found for size of the IA at time of rupture (Table 3).

### Sensitivity analyses

In the subgroup analyses only including studies defining familial IAs as at least two first-degree relative with IA (S3 and S4 Tables), only including high quality studies the results (S5 and S6 Tables) and excluding Finish and Inuit populations remained essentially the same (S7 and S8 Tables).

## Discussion

In familial patients IAs are more often located at the MCA and more often multiple. These findings were consistent across the studies. Familial aneurysms also seem to rupture at younger age than non-familial aneurysms, but these data showed high heterogeneity between the included studies and should therefore be interpreted carefully. No relevant differences between familial IA and non-familial IA patients were found for the characteristics sex, smoking, hypertension, size at time of rupture.

The analysis of ruptured IAs only showed a higher prevalence of IAs in the MCA in familial IA patients compared to non-familial patients. The analysis of ruptured IAs and unruptured IAs showed a comparable result, but this difference was not statistically significant. Although IAs in the MCA are more prevalent in familial patients, they are still relatively common in sporadic patients as well. Therefore, the presence of an MCA aneurysm cannot be interpreted as a risk factor for familial IA in clinical practice. Interestingly, a genetic study has established that genetic risk variants for IAs are enriched in patients with IAs located at the MCA suggesting that genetic risk factors play a more important role in the development of IAs at the MCA as compared to IAs at other sites at the circle of Willis [26].

In the comparison of the mean size of the IA at time of rupture the size tended to be lower in the familial IA group (12.9 mm) compared with that in the non-familial IA group (14.7 mm), but we were not able to demonstrate this difference with statistical significance. The mean sizes of rupture in both the familial and non-familial IA group are remarkably high (12.9 mm and 14.7 mm) considering the mean size of aneurysmal rupture is estimated around 6 mm [27]. These large mean sizes are mainly driven by a study in the Finnish population with a mean size at time of rupture of 17.2mm in 120 familial patients and of 22.0 mm in 1733 non-familial patients [21]. In comparison other reported sizes at time of rupture range from 10.5 to 11.0 to for familial patients and 8.1 to 14.9 to for sporadic patients [10,14].

This study is subject to several limitations. First, due to differences in reported measures of size of the IA and age at time of rupture we were unable to use all available evidence on these characteristics for our meta-analysis. For age at time of rupture one study could not be included as it was the only one reporting the median age at rupture while others reported the mean age instead [3, 6, 7, 10–14, 22]. For the analysis on size at time of rupture we had to exclude one study that reported median size instead of mean size and another study reporting the number of IAs per size range [7,13]. Due to differences in reported measures on condition on admission and outcome these characteristics could not be compared at all in our meta-analysis. Second, the definition of familial IAs varied across studies. However, the sensitivity analysis in which only studies with the strictest definition of familial IAs (defined as at least two first-degree relatives with IAs) were included showed comparable results to the main analysis. Third, some studies have compared familial IA patients to series of controls in whom a history of IAs in the family has not been excluded while in other studies exclusion of the presence of familial IAs has only occurred through interview, and not through analysis of medical records and/or screening relatives with angiography [11, 4]. Consequently, in these studies patients with familial IAs may have erroneously been included as having non-familial IAs potentially leading to an underestimation of the effect sizes of found associations. Fourth, smoking, hypertension and size at aneurysm rupture were only reported in the minority of publications used in this meta-analysis. Therefore, results of these comparisons must be interpreted with some caution. Furthermore, smoking and hypertension were only available from studies analyzing ruptured and unruptured IAs together or unruptured IAs only, and could thus not be assessed in the analysis on ruptured IAs only. Furthermore, a high percentage of patients included in this study is Finnish and the period of inclusion is heterogeneous which may limit the generalizability of the results. A higher incidence of aSAH and different patient and aneurysm characteristics have been reported for Finnish patients [28, 29,30], however in our subgroup analysis excluding Finnish (and Inuit) populations results remained essentially the same. Therefore, we do not believe that inclusion of these patients has influenced our findings to large extent.

## Conclusion

The results of this meta-analysis suggest that characteristics of familial and non-familial IAs show considerable overlap, yet differ on specific aspects. This should be taken into consideration for future etiological research into IAs. Further studies into differences in the risk of rupture and outcome between familial and non-familial IA are necessary to be able to draw more robust conclusions regarding the clinical implications of a positive family history in IA.

## Supporting information

S1 Checklist PRISMA Checklist. (DOC)

S1 Dataset Dataset systematic review. (SAV)

S1 File Full electronic search strategy. (DOCX)

S2 File Assessment of publication bias. (DOCX)

S3 File Forest plots. (DOCX)

S1 Table Definition of familial intracranial aneurysms and method of diagnosis. (DOCX)

S2 Table Included studies with enrolment period, extracted data and study quality score. (DOCX)

S3 Table Sensitivity analysis strict familial IA definition. Results of the comparison of patient and aneurysm-specific characteristics for ruptured and unruptured aneurysms.
(DOCX)

S4 Table Sensitivity analysis strict familial IA definition. Results of the comparison of patient and aneurysm-specific characteristics for ruptured aneurysms only.
(DOCX)

S5 Table Sensitivity analysis high quality studies only. Results of the comparison of patient and aneurysm-specific characteristics for ruptured and unruptured aneurysms.
(DOCX)

S6 Table Sensitivity analysis high quality studies only. Results of the comparison of patient and aneurysm-specific characteristics for ruptured aneurysms only.
(DOCX)

S7 Table Sensitivity analysis excluding Finnish and Inuit populations. Results of the comparison of patient and aneurysm-specific characteristics for ruptured and unruptured aneurysms.
(DOCX)

S8 Table Sensitivity analysis excluding Finnish and Inuit populations. Results of the comparison of patient and aneurysm-specific characteristics for ruptured aneurysms only.
(DOCX)
