# Supplementary material for: Patient and aneurysm characteristics in familial intracranial aneurysms. A systematic review and meta-analysis
Source: PLoS One. 2019 Apr 8;14(4):e0213372. doi: 10.1371/journal.pone.0213372 (PMC6453525; doi:10.1371/journal.pone.0213372)
Supplement: S1 File — (DOCX) [file pone.0213372.s003.docx]

**Full Electronic Search Strategy**

**PubMed**

((“Familial”[Title/Abstract] OR “Family”[Title/Abstract] OR “Families”[Title/Abstract] OR “Heritable”[Title/Abstract] OR “Heredity”[Title/Abstract] OR “First-degree Relatives”[Title/Abstract] OR “Genetic”[Title/Abstract])) AND (“Intracranial Aneurysm”[Title/Abstract] OR “Cerebral Aneurysm”[Title/Abstract] OR “Subarachnoid Hemorrhage”[Title/Abstract] OR “ Subarachnoid Haemorrhage”[Title/Abstract])

Results December 8^th^ 2018: 941

**EMBASE**

('familial':ab,ti OR 'family':ab,ti OR 'families':ab,ti OR 'heritable':ab,ti OR 'heredity':ab,ti OR 'first-degree relatives':ab,ti OR 'genetic':ab,ti) AND ('intracranial aneurysm':ab,ti OR 'cerebral aneurysm':ab,ti OR 'subarachnoid hemorrhage':ab,ti OR 'subarachnoid haemorrhage':ab,ti) AND [embase]/lim

Results December 8^th^ 2018: 1186
