# Supplementary material for: Patient and aneurysm characteristics in familial intracranial aneurysms. A systematic review and meta-analysis
Source: PLoS One. 2019 Apr 8;14(4):e0213372. doi: 10.1371/journal.pone.0213372 (PMC6453525; doi:10.1371/journal.pone.0213372)

**Forest plots of multiplicity of aneurysms, age at rupture and MCA aneurysms**

**Fig 1. Forest plot Multiplicity of Aneurysms.**


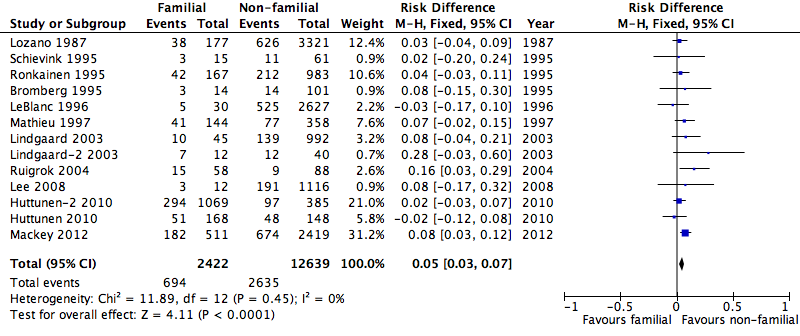


**Fig 2. Forest plot MCA Aneurysms.**


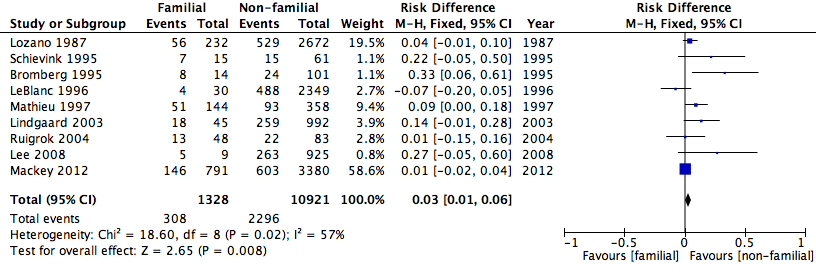


**Fig 3. Forest plot Age at Rupture.**


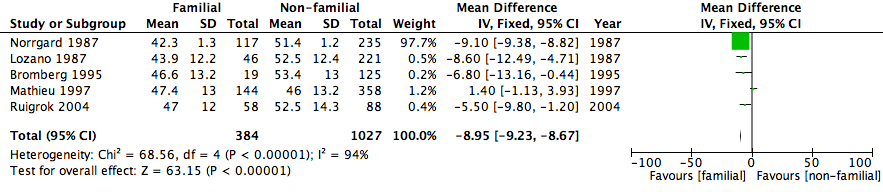

Supplement: S3 File — (DOCX) [file pone.0213372.s005.docx]
