# Supplementary material for: Patient and aneurysm characteristics in familial intracranial aneurysms. A systematic review and meta-analysis
Source: PLoS One. 2019 Apr 8;14(4):e0213372. doi: 10.1371/journal.pone.0213372 (PMC6453525; doi:10.1371/journal.pone.0213372)
Supplement: S1 Table — (DOCX) [file pone.0213372.s006.docx]

**Supporting Information 1 Table**

**Definition of familial intracranial aneurysms and method of diagnosis.**

| **First Author** | **Definition of familial IA** | **Method of Diagnosis**^a^ |
| --- | --- | --- |
| **Lozano^11^** | ≥ 2 family members | Records |
| **Norrgard^10^** | ≥ 2 family members | Interview, records |
| **Ronkainen^3^** | ≥ 2 first-degree family members | Interview |
| **Bromberg^9^** | ≥ 2 first-degree family members | Interview and records |
| **Schievink^6^** | ≥ 2 first-degree family members | Interview and records |
| **LeBlanc^4^** | ≥ 2 family members | Records |
| **Mathieu^7^** | ≥2 first-to-third degree family members | Records |
| **Ronkainen^21b^** | ≥ 2 first-degree family members | Interview |
| **Connolly^8^** | ≥ 2 first-degree family members^c^ | Interview, records, angiography |
| **Lindgaard^12^** | ≥ 2 first- or second-degree family members | Records |
| **Ruigrok^14^** | ≥ 2 first-degree family members | Interview, records, angiography |
| **Lee^22^** | ≥ 2 first-degree family members | Interview and records |
| **Broderick^20^** | ≥ 2 first-degree family members | Records and angiography |
| **Huttunen^13^** | ≥ 2 first-degree family members | Interview |
| **Mackey^5^** | ≥3 family members or ≥2 siblings | Interview, records, angiography |

IA= intracranial aneurysm

^a^Method of diagnosis used: interview, medical records or screening with imaging or a combination

^b^This cohort is largely overlapping with Ronkainen et al. 1995^3^
^c^Only sibling pairs were analyzed
