# Supplementary material for: Patient and aneurysm characteristics in familial intracranial aneurysms. A systematic review and meta-analysis
Source: PLoS One. 2019 Apr 8;14(4):e0213372. doi: 10.1371/journal.pone.0213372 (PMC6453525; doi:10.1371/journal.pone.0213372)
Supplement: S2 Table — (DOCX) [file pone.0213372.s007.docx]

**Supporting Information 2 Table**

**Table 2. Included studies with enrolment period, extracted data and study quality score.**

| **First Author** | **Enrolment period** | **Extracted patient characteristics** | **Extracted aneurysm characteristics** | **Study quality^a^** |
| --- | --- | --- | --- | --- |
| **Lozano^11^** | Before 1985 | Sex, age at rupture | Multiplicity, mean size at rupture, location (ACA, MCA, ICA, VBA) | 3 |
| **Norrgard^10^** | 1969-1981 | Sex, age at rupture | Multiplicity, location (ACA, MCA, ICA, VBA) | 6 |
| **Ronkainen^3^** | 1977-1990 | Sex, age at rupture | Multiplicity, location (ACA, MCA, ICA, VBA) | 6 |
| **Bromberg^9^** | 1991-1992 | Sex, age at rupture | Multiplicity, location (ACA, MCA, ICA) | 6 |
| **Schievink^6^** | 1970-1989 | Sex, age at rupture | Multiplicity, location (ACA, MCA, ICA, VBA) | 4 |
| **LeBlanc^4^** | 1986-1997 | Sex | Multiplicity, location (ACA, ICA, MCA, VBA) | 3 |
| **Mathieu^7^** | 1965-1993 | Sex, age at rupture | Multiplicity, location (ACA, MCA, ICA) | 6 |
| **Ronkainen^21b^** | 1977-1995 | None | Mean size at rupture | 6 |
| **Connolly^8^** | 1986-1995 | Sex, smoking, hypertension | None | 3 |
| **Lindgaard^12^** | 1978-1998 | Sex, age at rupture | Multiplicity, location (ACA, MCA, ICA, VBA) | 4 |
| **Ruigrok^14^** | 1995-1997 | Sex, age at rupture | Multiplicity, mean size at rupture, location (ACA, MCA, ICA, VBA) | 6 |
| **Lee^22^** | 1993-2006 | Sex, age at rupture | Multiplicity, location (ACA, MCA, ICA) | 4 |
| **Broderick^20^** | - | Sex, hypertension | Multiplicity | 6 |
| **Huttunen^13^** | 1993-2007 | Sex | Multiplicity, location (ACA, MCA, ICA, VBA) | 6 |
| **Mackey^5^** | - | Sex, smoking, hypertension | Multiplicity, location (ACA, MCA, ICA, VBA) | 4 |

ACA= anterior cerebral artery, including the anterior communicating artery and pericallosal artery, MCA= medial cerebral artery, ICA= internal carotid artery, VBA= vertebrobasilar artery

^a^Score based on Newcastle Ottawa Scale

^b^This cohort is largely overlapping with Ronkainen et al. 1995^3^
