# Supplementary material for: Patient and aneurysm characteristics in familial intracranial aneurysms. A systematic review and meta-analysis
Source: PLoS One. 2019 Apr 8;14(4):e0213372. doi: 10.1371/journal.pone.0213372 (PMC6453525; doi:10.1371/journal.pone.0213372)
Supplement: S3 Table — Results of the comparison of patient and aneurysm-specific characteristics for ruptured and unruptured aneurysms. (DOCX) [file pone.0213372.s008.docx]

**Supporting Material 3 Table**

**Sensitivity analysis strict familial IA definition.**

| **Characteristic** | **Familial IAs** | **Non-familial IAs** | **Β^a^** | **95% CI** | **P-value** | **Heterogeneity**  **I^2^ (%)** |
| --- | --- | --- | --- | --- | --- | --- |
| **Women (%)** | 58.5 | 63.0 | -0.04 | -0.17-0.06 | 0.55 | 40 |
| **Multiplicity (%)** | 27.0 | 19.1 | 0.10 | 0.03-0.17 | 0.007 | 0 |

Results of the comparison of patient and aneurysm-specific characteristics for ruptured and unruptured aneurysms.

IA=intracranial aneurysm, 95% CI=95% confidence interval

^a^beta calculated with weighted linear regression
