# Supplementary material for: Patient and aneurysm characteristics in familial intracranial aneurysms. A systematic review and meta-analysis
Source: PLoS One. 2019 Apr 8;14(4):e0213372. doi: 10.1371/journal.pone.0213372 (PMC6453525; doi:10.1371/journal.pone.0213372)
Supplement: S4 Table — Results of the comparison of patient and aneurysm-specific characteristics for ruptured aneurysms only. (DOCX) [file pone.0213372.s009.docx]

**Supporting Material 4 Table**

**Sensitivity analysis strict familial IA definition.**

| **Characteristic** | **Familial IAs** | **Non-familial IAs** | **Β^a^** | **95% CI** | **P-value** | **Heterogeneity**  **I^2^ (%)** |
| --- | --- | --- | --- | --- | --- | --- |
| **Women (%)** | 57.5 | 62.9 | -0.05 | -0.23-0.14 | 0.59 | 46 |
| **Age at rupture (yrs.)** | 48.6 | 53.0 | -5.21 | -12.63-1.60 | 0.12 | 0 |
| **ACA (%)** | 22.4 | 38.2 | 0.03 | -0.27-0.33 | 0.84 | 78 |
| **ICA (%)** | 23.4 | 24.2 | -0.02 | -0.06-0.02 | 0.33 | 0 |
| **MCA (%)** | 45.4 | 28.9 | 0.10 | -0.01-0.21 | 0.07 | 33 |
| **VBA (%)** | 6.8 | 8.3 | -0.03 | -0.10-0.05 | 0.46 | 0 |

Results of the comparison of patient and aneurysm-specific characteristics for ruptured aneurysms only.

IA=intracranial aneurysm, 95% CI=95% confidence interval, ACA= anterior cerebral artery, including the anterior communicating artery and pericallosal artery, MCA= medial cerebral artery, ICA= internal carotid artery, VBA= vertebrobasilar artery

^a^beta calculated with weighted linear regression
