# Supplementary material for: Patient and aneurysm characteristics in familial intracranial aneurysms. A systematic review and meta-analysis
Source: PLoS One. 2019 Apr 8;14(4):e0213372. doi: 10.1371/journal.pone.0213372 (PMC6453525; doi:10.1371/journal.pone.0213372)
Supplement: S5 Table — Results of the comparison of patient and aneurysm-specific characteristics for ruptured and unruptured aneurysms. (DOCX) [file pone.0213372.s010.docx]

**Supporting Material 5 Table**

**Sensitivity analysis high quality studies only.**

| **Characteristic** | **Familial** | **Non-familial** | **Β**^a^ | **95% CI** | **P-value** | **Heterogeneity**  **I^2^(%)** |
| --- | --- | --- | --- | --- | --- | --- |
| **Women (%)** | 60.2 | 56.7 | 0.04 | -0.06-0.15 | 0.38 | 71 |
| **Multiplicity (%)** | 28.5 | 20.0 | 0.08 | 0.02-0.14 | 0.01 | 0 |
| **ACA (%)** | 28.8 | 35.3 | -0.03 | -0.17-0.12 | 0.70 | 63 |
| **ICA (%)** | 18.6 | 21.4 | -0.01 | -0.11-0.09 | 0.79 | 0 |
| **MCA (%)** | 42.5 | 31.3 | 0.07 | -0.03-0.18 | 0.15 | 7 |
| **VBA (%)** | 4.8 | 6.5 | -0.02 | -0.10-0.06 | 0.52 | 3 |

Results of the comparison of patient and aneurysm-specific characteristics for ruptured and unruptured aneurysms.

IA=intracranial aneurysm, 95% CI=95% confidence interval, ACA= anterior cerebral artery, including the anterior communicating artery and pericallosal artery, MCA= medial cerebral artery, ICA= internal carotid artery, VBA= vertebrobasilar artery

^a^beta calculated with weighted linear regression
