# Supplementary material for: Patient and aneurysm characteristics in familial intracranial aneurysms. A systematic review and meta-analysis
Source: PLoS One. 2019 Apr 8;14(4):e0213372. doi: 10.1371/journal.pone.0213372 (PMC6453525; doi:10.1371/journal.pone.0213372)
Supplement: S6 Table — Results of the comparison of patient and aneurysm-specific characteristics for ruptured aneurysms only. (DOCX) [file pone.0213372.s011.docx]

**Supporting Material 6 Table**

**Sensitivity analysis high quality studies only.**

| **Characteristic** | **Familial** | **Non-familial** | **Β**^a^ | **95% CI** | **P-value** | **Heterogeneity**  **I^2^(%)** |
| --- | --- | --- | --- | --- | --- | --- |
| **Women (%)** | 59.9 | 56.8 | 0.03 | -0.10-0.17 | 0.60 | 75 |
| **Age at rupture (yrs.)** | 46.3 | 50.8 | -2.87 | -7.12-1.39 | 0.16 | 82 |
| **Size at rupture (mm)** | 14.1 | 14.6 | -5.05 | 35.18-25.08 | 0.55 | 99 |
| **ACA (%)** | 28.8 | 35.3 | -0.03 | -0.17-0.12 | 0.70 | 63 |
| **ICA (%)** | 18.6 | 21.4 | -0.01 | -0.11-0.09 | 0.79 | 0 |
| **MCA (%)** | 42.5 | 31.3 | 0.07 | -0.03-0.18 | 0.15 | 7 |
| **VBA (%)** | 4.8 | 6.5 | -0.02 | -0.10-0.06 | 0.52 | 3 |

Results of the comparison of patient and aneurysm-specific characteristics for ruptured aneurysms only.

IA=intracranial aneurysm, 95% CI=95% confidence interval, ACA= anterior cerebral artery, including the anterior communicating artery and pericallosal artery, MCA= medial cerebral artery, ICA= internal carotid artery, VBA= vertebrobasilar artery

^a^beta calculated with weighted linear regression
