# Supplementary material for: Patient and aneurysm characteristics in familial intracranial aneurysms. A systematic review and meta-analysis
Source: PLoS One. 2019 Apr 8;14(4):e0213372. doi: 10.1371/journal.pone.0213372 (PMC6453525; doi:10.1371/journal.pone.0213372)
Supplement: S7 Table — Results of the comparison of patient and aneurysm-specific characteristics for ruptured and unruptured aneurysms. (DOCX) [file pone.0213372.s012.docx]

**Supporting Material 7 Table**

**Sensitivity analysis excluding Finnish and Inuit populations.**

| **Characteristic** | **Familial** | **Non-familial** | **Β**^a^ | **95% CI** | **P-value** | **Heterogeneity**  **I^2^(%)** |
| --- | --- | --- | --- | --- | --- | --- |
| **Women (%)** | 65.1 | 63.7 | 0.056 | 0.056-0.169 | 0.310 | 68 |
| **Smoking** | 55.7 | 52.6 | -0.056 | -0.888-0.775 | 0.798 | 72 |
| **Hypertension** | 47.5 | 54.9 | 0.009 | -0.457-0.475 | 0.956 | 95 |
| **Multiplicity (%)** | 25.2 | 17.9 | 0.113 | 0.041-0.185 | 0.004 | 0 |
| **ACA (%)** | 20.7 | 33.4 | -0.072 | -0.268-0.124 | 0.450 | 55 |
| **ICA (%)** | 29.1 | 26.8 | -0.028 | -0.162-0.105 | 0.659 | 16 |
| **MCA (%)** | 36.7 | 25.5 | 0.028 | -0.058-0.133 | 0.503 | 44 |
| **VBA (%)** | 6.8 | 6.6 | 0.006 | -0.039-0.051 | 0.776 | 0 |

Results of the comparison of patient and aneurysm-specific characteristics for ruptured aneurysms and unruptured aneurysms.

IA=intracranial aneurysm, 95% CI=95% confidence interval, ACA= anterior cerebral artery, including the anterior communicating artery and pericallosal artery, MCA= medial cerebral artery, ICA= internal carotid artery, VBA= vertebrobasilar artery

^a^beta calculated with weighted linear regression
