# Supplementary material for: Patient and aneurysm characteristics in familial intracranial aneurysms. A systematic review and meta-analysis
Source: PLoS One. 2019 Apr 8;14(4):e0213372. doi: 10.1371/journal.pone.0213372 (PMC6453525; doi:10.1371/journal.pone.0213372)
Supplement: S8 Table — Results of the comparison of patient and aneurysm-specific characteristics for ruptured aneurysms only. (DOCX) [file pone.0213372.s013.docx]

**Supporting Material 8 Table**

**Sensitivity analysis excluding Finnish and Inuit populations.**

| **Characteristic** | **Familial** | **Non-familial** | **Β**^a^ | **95% CI** | **P-value** | **Heterogeneity**  **I^2^(%)** |
| --- | --- | --- | --- | --- | --- | --- |
| **Women (%)** | 64.6 | 61.7 | 0.036 | -0.110-0.182 | 0.602 | 60 |
| **Age at rupture (yrs.)** | 46.8 | 51.8 | -5.775 | -11.311- -0.238 | 0.042 | 99 |
| **Multiplicity (%)** | 22.8 | 16.4 | 0.071 | -0.005-0.146 | 0.064 | 0 |
| **Size at rupture (mm)** | 10.8 | 11.5 | 0.024 | -16.451-16.500 | 0.995 | 99 |
| **ACA (%)** | 21.9 | 36.3 | -0.002 | -0.282-0.278 | 0.990 | 48 |
| **ICA (%)** | 27.4 | 25.1 | -0.045 | -0.246-0.157 | 0.642 | 0 |
| **MCA (%)** | 40.5 | 27.2 | 0.122 | -0.005-0.250 | 0.059 | 28 |
| **VBA (%)** | 6.9 | 6.7 | 0.011 | -0.069-0.092 | 0.750 | 0 |

Results of the comparison of patient and aneurysm-specific characteristics for ruptured aneurysms only.

IA=intracranial aneurysm, 95% CI=95% confidence interval, ACA= anterior cerebral artery, including the anterior communicating artery and pericallosal artery, MCA= medial cerebral artery, ICA= internal carotid artery, VBA= vertebrobasilar artery

^a^beta calculated with weighted linear regression
